# Antioxidant rich grape pomace extract suppresses postprandial hyperglycemia in diabetic mice by specifically inhibiting alpha-glucosidase

**DOI:** 10.1186/1743-7075-7-71

**Published:** 2010-08-27

**Authors:** Shelly Hogan, Lei Zhang, Jianrong Li, Shi Sun, Corene Canning, Kequan Zhou

**Affiliations:** 1 Montana State University, Bozeman, Montana, 59717, USA; 2 College of Food Science, Biotechnology and Environmental Engineering, Zhejiang Gongshang University, Hangzhou, 310035, China; 3 Department of Nutrition and Food Science, Wayne State University, Detroit, MI, 48202, USA

## Abstract

**Background:**

Postprandial hyperglycemia is an early defect of type 2 diabetes and one of primary anti-diabetic targets. Treatment of postprandial hyperglycemia can be achieved by inhibiting intestinal α-glucosidase, the key enzyme for oligosaccharide digestion and further glucose absorption. Grape pomace is winemaking byproduct rich in bioactive food compounds such as phenolic antioxidants. This study evaluated the anti-diabetic potential of two specific grape pomace extracts by determining their antioxidant and anti-postprandial hyperglycemic activities *in vitro *and *in vivo*.

**Methods:**

The extracts of red wine grape pomace (Cabernet Franc) and white wine grape pomace (Chardonnay) were prepared in 80% ethanol. An extract of red apple pomace was included as a comparison. The radical scavenging activities and phenolic profiles of the pomace extracts were determined through the measurement of oxygen radical absorbance capacity, DPPH radical scavenging activity, total phenolic content and flavonoids. The inhibitory effects of the pomace extracts on yeast and rat intestinal α-glucosidases were determined. Male 6-week old C57BLKS/6NCr mice were treated with streptozocin to induce diabetes. The diabetic mice were then treated with vehicle or the grape pomace extract to determine whether the oral intake of the extract can suppress postprandial hyperglycemia through the inhibition of intestinal α-glucosidases.

**Results:**

The red grape pomace extract contained significantly higher amounts of flavonoids and phenolic compounds and exerted stronger oxygen radical absorbance capacity than the red apple pomace extract. Both the grape pomace extracts but not the apple pomace extract exerted significant inhibition on intestinal α-glucosidases and the inhibition appears to be specific. In the animal study, the oral intake of the grape pomace extract (400 mg/kg body weight) significantly suppressed the postprandial hyperglycemia by 35% in streptozocin-induced diabetic mice following starch challenge.

**Conclusion:**

This is the first report that the grape pomace extracts selectively and significantly inhibits intestinal α-glucosidase and suppresses postprandial hyperglycemia in diabetic mice. The antioxidant and anti-postprandial hyperglycemic activities demonstrated on the tested grape pomace extract therefore suggest a potential for utilizing grape pomace-derived bioactive compounds in management of diabetes.

## Background

The prevalence of diabetes has risen at an alarming rate. Considering the heterogeneity of diabetes and the limitations of current therapies, such as high secondary failure rates and significant side effects, there is an urgent need to explore novel health-promotion and therapeutical strategies. One intriguing approach to control diabetes could be its prevention and treatment by phytochemicals present in the diet that improve postprandial glycemic control and reduce postprandial hyperglycemia [1-4]. Postprandial hyperglycemia is an early detected symptom in type 2 diabetes [5], which occurs when pancreatic β cells fail to secrete a sufficient amount of insulin [6]. Emerging evidence suggests that postprandial hyperglycemia induces glucose toxicity and deteriorates β cell function [7], which can ultimately result in an irreversible state of diabetes [8].

Postprandial hyperglycemia is related to the amount and digestion rate of consumed starch, which is the primary source of blood glucose. One important approach for treating postprandial hyperglycemia is to reduce or slow dietary carbohydrate digestion and absorption [9-11]. This approach can be achieved by inhibiting starch hydrolyzing enzymes in the digestive system [12-14]. Mammalian starch digestion primarily occurs in the small intestine through the action of α-amylase, yielding both linear maltose and branched isomaltose oligosaccharides, which are further hydrolyzed by α-glucosidases to release glucose [15]. Synthetic and naturally derived compounds are known to reduce postprandial hyperglycemia by inhibiting key carbohydrate metabolizing enzyme in the small intestine such as α-glucosidase [16,17]. For example, phenolic compounds comprised in plants have been found to be potent inhibitors of carbohydrate hydrolyzing enzymes [18-21]. In a screening test among more than 300 food extracts and compounds, we identified a grape pomace extract exert significant inhibition of α-glucosidases, suggesting its potential use for diabetes management.

Grape pomace is rich in phenolic compounds but typically regarded as a waste byproduct generated in the lucrative winemaking industry [22]. Large amounts of this byproduct accumulate annually which leads to a waste-management issue [23]. It is estimated that the harvested grapes will generate approximately 20% of grape pomace. However, uses of grape pomace are limited but have been recycled as organic fertilizers, manure, and animal feed. On the other hand, grapes are known to be a natural source of notable bioactive compounds in particular antioxidants with potential health promoting and disease protective qualities [24-26]. For example, wine, grapes, and grape seed extracts are a major source of polyphenolic components such as anthocyanins, flavanols, catechins, and proanthocyanidins [27-31]. Because grape skins and seeds are the predominant constituents in the pomace, this biomass is speculated to be a rich source of antioxidants [32,33]. Dietary antioxidants have been associated with the reduced risk of type 2 diabetes by inhibiting peroxidation chain reactions. However, very few studies have investigated the potential of grape pomace as an alternative bioresource for diabetes management [22,33-38].

The aim of this study was to evaluate anti-diabetic potential of the grape pomace extracts by determining their antioxidant and anti-postprandial hyperglycemic activities. Two grape pomace extracts: one red wine grape pomace (Cabernet Franc, RGPE) and one white wine grape pomace (Chardonnay, WGPE) were investigated for their total phenolic contents, total flavonoids, free radical scavenging activities, and their inhibitory effects against yeast and rat intestinal α-glucosidase. We also included an extract of a red apple pomace (RAPE) for comparison because the apple pomace is also rich in phenolic antioxidants but its bioactivities may be different.

## Methods

### Materials

Yeast type I α-glucosidase (EC 3.2.1.20, G5003), rat intestinal acetone powder (N1377-5G), *p*-nitrophenyl α-D-glucoside (pNPG), acarbose, porcine pancreatic α-amylase, type VI-B (A3176), porcine pancreatic lipase, Type ll (L3216), Folin-Ciocalteu reagent, gallic acid, rutin, Trolox™, fluorescein, 2,2'-Azobis(2-amidinopropane) dihydrochloride (AAPH), 1,1-diphenyl-2-picrylhydrazyl (DPPH), and streptozotocin (STZ) were purchased from the Sigma Chemical Co. (St. Louis, MO, USA). The ethanol and acetone solvent was HPLC grade (Fisher Scientific Co.).

### Preparation of pomace extracts

Red wine grape pomace (Cabernet Franc) and white wine grape pomace (Chardonnay) were obtained from a local Virginia vineyard (Blackstone, VA, USA). The red apple pomace was obtained from National fruit product company, INC. (Winchester, VA). All the pomace extracts were prepared from a single production lot. A portion of the pomace samples (500 g) were immediately freeze-dried upon receiving. The dried extracts were then ground to fine powder by a Thomas Wiley mini-mill (Swedesboro, NJ). The samples were extracted with 80% ethanol at 1:10 ratio (m/v) under overnight shaking. The extracts were filtered through Whatman No. 4 filter paper to remove unwanted residues. After evaporating off the organic solvent, the filtrates were frozen and lyophilized to obtain the pomace extracts. The extraction yield was 13.4%, 16.3%, and 13.8% for the dried red grape, white grape, and red apple pomace, respectively. A portion of the lyophilized extracts were freshly reconstituted in dimethyl sulfoxide (DMSO) at 20 mg/mL as the stock solution and stored at -20°C for further investigation.

### Total phenolic content (TPC)

The TPC of each pomace extract was determined using Folin-Ciocalteu reagent with gallic acid as the phenolic standard [39]. In brief, appropriate dilutions of extracts were mixed with 3.0 mL of 0.2 N Folin-Ciocalteu reagent and 2.0 mL of 20% sodium carbonate (Na_2_CO_3_) at ambient temperature. After incubation for 2 hours, the absorbance of the blue color that developed in each assay mixture was recorded at 760 nm (Thermo Electron Corporation, Genesys 10-UV scanning, Madison, WI, USA). The TPC value of each pomace extract was expressed as micrograms of gallic acid equivalent per gram of pomace (μg GAE/g).

### Total flavonoid content (TFC)

The pomace extracts were analyzed for TFC according to an established colormetric method [40]. In brief, 1 mL of the reconstituted pomace extract (500 μg/mL) or rutin standard was mixed with 0.3 mL of 5% sodium nitrite (NaNO_2_), 0.3 mL of 10% aluminum chloride (AlCl_3_), and 2 mL of 1 M sodium hydroxide (NaOH). The reaction mixture was incubated at 30°C for 30 min. All samples were measured in duplicate and compared against a blank at an absorbance of 510 nm (Thermo Electron Corporation, Genesys 10-UV scanning, Madison, WI, USA). Results were expressed as micrograms of rutin equivalent per gram of pomace (μg RE/g).

### Oxygen radical absorbance capacity (ORAC)

The ORAC assay was conducted to kinetically measure the peroxyl radical scavenging activity of each pomace extract with Trolox™ as the antioxidant standard (Zhou et al., 2007). Fluorescein (FL) was used as the fluorescent probe and the peroxyl radicals were generated from AAPH in 75 mM phosphate buffer (pH 7.4). Specifically, 225 μL of 81.6 nM FL solution was mixed with 30 μL of sample extract, standard, or blank (DMSO) to a black 96-well fat bottom plate and incubate covered plate at 37°C for 20 min. After incubation, 25 μL of 0.36 M APPH solution was added to the mixture and the reaction started. Standards and samples were measured in duplicate. The fluorescence of the reaction mixture was monitored and recorded every minute (λex = 485 nm and λem = 535 nm) and maintained at 37°C until the reading had declined to less than 5% of the initial reading with a Victor^3 ^multilabel plate reader (Perkin-Elmer, Turku, Finland). Results for ORAC were determined by using a regression equation relating Trolox™ concentrations and the net area under the kinetic fluorescein decay curve. The ORAC value was expressed in micromoles of Trolox™ equivalents per gram of pomace (μmol TE/g).

### DPPH radical scavenging activity

The DPPH radical scavenging antioxidant activity assay was conducted to obtain the antioxidant activity of pomace extracts [41]. The reaction mixture contained 100 μL of the diluted pomace extracts (1 mg/mL) and 100 μL of 0.208 mM DPPH radical solution. The absorption at 515 nm was determined immediately after the reaction was initiated. Each plate was read once every minute for 30 min with a Victor^3 ^multilabel plate reader. The initial and final absorbance for the control was 0.934 and 0.917, respectively. The percent inhibition of the DPPH radical scavenging activity per milligram of pomace extract was expressed as the inhibition percentage (% DPPH inhibition/mg).

### Yeast and mammalian α-glucosidase inhibition assays

Both the yeast and mammalian α-glucosidase activity was assayed using the substrate p-nitrophenyl-α-d-glucopyranoside (pNPG), which is hydrolyzed by α-glucosidase to release the product p-nitrophenol, a color agent that can be monitored at 405 nm [42]. The mammalian α-glucosidases were prepared from 1 g of rat intestinal acetone powder suspended in 20 mL of 0.1 M potassium phosphate buffer (pH 7.0) containing 5 mM EDTA at ambient temperature. The suspension was sonicated for 15 min and after vigorous stirring for 1 h, the suspension was centrifuged. The supernatant was dialyzed against 0.01 M potassium phosphate buffer (pH 7.0) for 24 hours. The activity of rat α-glucosidase extract was verified using pNPG as the substrate by comparing with the pure yeast α-glucosidase. The assays were conducted by mixing 80 μL of approximate dilutions of the pomace extracts (10 μg/mL) in 0.1 M phosphate buffer (pH 6.8) with 20 μL of the yeast enzyme solution (1 U/mL) or 20 μL rat intestinal α-glucosidase solution (3 × dilution from the original extract). The acarbose (150 μg/mL) was used as a positive control. The pH of the sample extracts were 6.8 which was the optimal for the enzyme reaction. The blank reagent, 0.1 M phosphate buffer (pH 6.8), was used as the control. The mixture was incubated in a 96-well plate at 37°C for 3 minutes under constant shaking. After incubation, 100 μL of 4 mM pNPG solution in 0.1 M phosphate buffer (pH 6.8) was added and the reaction was conducted at 37°C. The release of p-nitrophenol from pNPG was monitored at 405 nm every minute for 75 minutes with a Victor^3 ^multilabel plate reader. The α-glucosidase activity was determined by measuring the area under the curve (0-75 minutes) for each sample and compared with that of the control (the blank reagent). The results were expressed as the percent of α-glucosidase inhibition.

### Animal experiments

#### Animals

Male 6-week old mice (C57BLKS/6NCr, National Cancer Institute, Frederick, MD, USA) were housed in groups of four mice per cage and maintained on a 12-hour light-dark cycle at 20°C to 22°C. The animals were acclimatized for a 2-week period before starting the experiment and had *ad libitum *access to food and water. The mice were maintained on rodent feed (Harlan Tekland Gobal Diets 2018 rodent diet containing 60% of calories from carbohydrate, 23% of calories from protein, and 17% of calories from fat; digestible energy of 3.4 Kcal/g, Madison WI, USA) for the duration of the experiment. Animal husbandry, care, and experimental procedures were conducted in compliance with the "Principles of Laboratory Animal Care" NIH guidelines, as approved by the Institutional Animal Care and Use Committee (IACUC) at Virginia Tech.

#### STZ induction of diabetes in mice

Diabetes was induced in 14-hour fasted 8-week old mice (25-27 g) by intraperitoneal injection of STZ dissolved in 10 mM sodium citrate buffer (pH 4.5) at a dose of 50 mg/kg body weight (bw). The STZ was dissolved in ice-cold citrate buffer protected from light and injected immediately to avoid STZ degradation. Five to seven days after STZ injection, mice with a fasting blood glucose (FBG) level higher than 126 mg/dL were considered to have diabetes and were randomly assigned to one of two groups (n = 8).

#### Oral RGPE treatment and starch challenge

The experiment was designed to determine the effect of acute RGPE intake on postprandial glycemic response in STZ-induced diabetic mice following a potato starch challenge. Diabetic mice were fasted for 14-hours in freshly cleaned cages with free access to water before the experiment. Ten mg of the lyophilized RGPE was suspended in 0.2 mL water (50 mg/mL) in a small centrifuge tube and vortexed vigorously. The dietary dose was calculated to be 400 mg/kg bw based on mouse weight of 25 g. Mice in the control group were given 0.2 mL of water by oral gavage. The treatment group were administered 0.2 mL of RGPE suspension (400 mg/kg bw) by oral gavage immediately after vortexing the suspension. After approximately 30 minutes post water or RGPE administration, 0.2 mL of potato starch suspension (2 g/kg bw) was administered to each mouse by gavage. Approximately 5 μL of whole blood samples were collected from the tail vein of each mouse. The blood samples were acquired at 0, 30, 60, and 120 minutes after the oral starch challenge. Blood glucose levels were measured with a blood glucometer and accompanying test strips (ACCU-CHEK Meter^®^, Roche Diagnostics, Kalamazoo, MI). The area under the glucose tolerance curve (AUC_0-120 min_) was calculated using a trapezoidal method [43]. The total antihyperglycemic response (AUC_0-120 min_) was expressed as mean ± standard deviation.

### Statistical analysis

The statistical significance comparing data between groups was assessed by one-way analysis of variance (ANOVA) followed by Duncan's multiple range post-hoc tests. Statistical analysis was performed using SPSS (Windows, Version Rel. 10.0.5, 1999, SPSS Inc., Chicago, IL). Statistical significance was declared when *P *< 0.05.

## Results

### TPC and TFC in the pomace extracts

As shown in Table 1, all the three tested pomace samples contained noticeable amount of phenolic and flavonoid compounds. The RGPE contained the highest TPC (30.4 mg GAE/g) followed by the WGPE (24.5 mg GAE/g), while the red apple pomace extract (RAPE) contained the least TPC (11.2 mg GAE/g). In a similar trend, the RGPE contained the highest TFC (22.1 mg RE/g), followed by the WGPE (16.2 mg RE/g) and the RAPE (5.7 mg RE/g). The RGPE contained significantly higher TPC and TFC than the RAPE. Flavonoids account for 72% and 66% of total phenolic compounds in the RGPE and WGPE, respectively. However, in the RAPE, only approximately 50% phenolic compounds are flavonoids, suggesting that the profile of phenolic compounds between grape and apple pomaces could be remarkably different.

**Table 1:** Total phenolic content, total flavonoid content, ORAC, and DPPH of the pomace extracts.

	Red grape pomace	White grape pomace	Red Apple pomace
Total phenolic content (mg GAE/g)	30.4 ± 11.0^a^	24.5 ± 6.0^a,b^	11.2 ± 5.9^b^
Total flavonoid content (mg RE/g)	22.1 ± 8.9^a^	16.2 ± 5.4^a,b^	5.7 ± 4.7^b^
ORAC (μmol TE/g)	245.3 ± 21.0^a^	197.7 ± 25.5^b^	168.8 ± 22.7^b^
%DPPH Inhibition/mg	66.1 ± 0.6	67.4 ± 4.1	54.6 ± 13.2

Total phenolic content is expressed as μg of gallic acid equivalents (GAE) per gram of pomace; total flavonoid content is expressed as μg of rutin equivalents (RE) per gram of pomace. Oxygen radical absorbance capacity (ORAC) was measured in triplicate, values expressed as micromoles Trolox equivalents per gram of pomace (μmol TE/g). The DPPH radical scavenging activity was expressed as percent inhibition of DPPH free radical per mg of pomace. For each measurement, the data marked by different letters for each assay are significantly different (P < 0.05).

### ORAC and DPPH radical scavenging activities of the pomace extracts

The ORAC assay measured the scavenging capacity of pomace extracts against peroxyl radicals. As shown in Table 1, the RGPE exerted the highest ORAC value (245.0 μmol TE/g) as compared to either the WGPE (198.3 μmol TE/g) or the RAPE (168.8 μmol TE/g, *P *< 0.05). One milligram of the RGPE, WGPE, and the RAPE quenched 66%, 67%, and 55% of DPPH radicals in the reaction, respectively. However, no statistical differences were detected between any of the three tested pomace extracts.

### *In vitro *yeast and rat intestinal α-glucosidase inhibition by the pomace extracts

**Figure 1:**
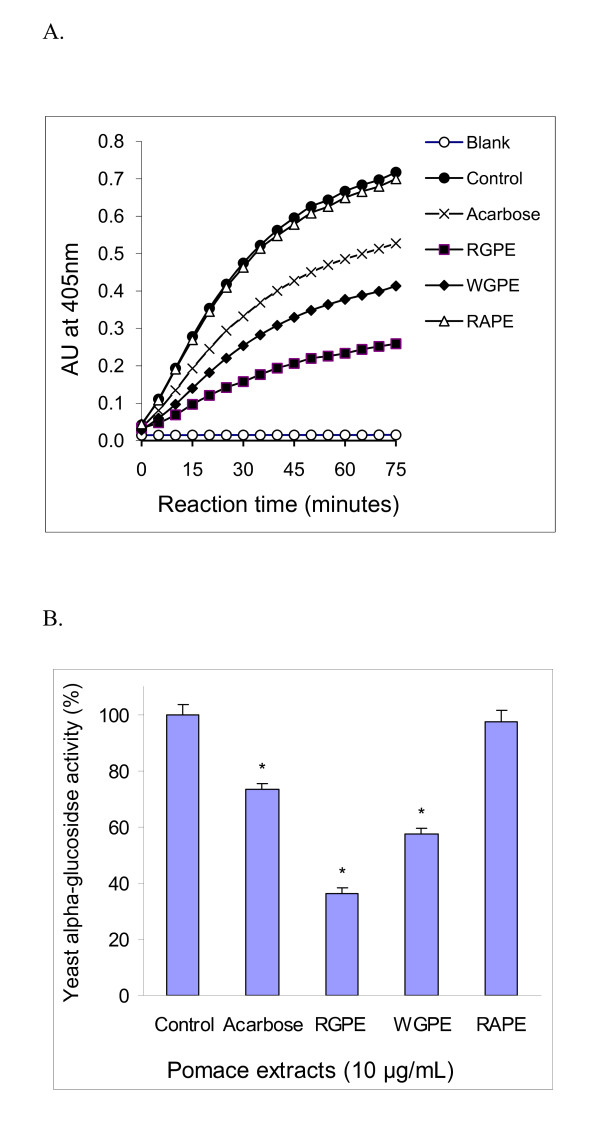
**The inhibitory effects of the grape and apple pomace extracts on yeast α-glucosidase activity: A. Time responses of the pomace extracts on the enzyme activity; B. Comparison of the inhibitory activities by the different pomace extracts**. Acarbose (150 μg/mL), is a commercially available oral alpha-glucosidase inhibitor which served as the positive control. The α-glucosidase activity was determined by measuring *p*-nitrophenol released from pNPG at 405 nm. The reaction was conducted at 37°C for 75 minutes. Results are expressed as mean ± standard deviation. * significant difference versus the control (*P *< 0.05).

Yeast α-glucosidase is readily available in a pure form and has been widely used for anti-diabetes nutraceutical and medicinal investigations as a model for screening potential inhibitors [13,20,42,44-46]. Figure 1A reveals the time-responses of the pomace extracts (10 μg/mL) against yeast α-glucosidase. Both the RGPE and WGPE significantly inhibited the enzyme activity and the inhibition was sustained during 75 minutes of the incubation. However, the RAPE showed no inhibition on yeast α-glucosidase, suggesting grape pomaces contain unique α-glucosidase inhibitory compounds. Interestingly, both the RGPE and WGPE showed stronger α-glucosidase inhibitory activity than acarbose, a commercial α-glucosidase inhibitor. As shown in Figure 1B, the RGPE and WGPE both at 10 μg/mL inhibited yeast α-glucosidase activity by 63.9% and 42.4%, respectively; while acarbose at 150 μg/mL only inhibited the enzyme activity by 26.5%.

**Figure 2:**
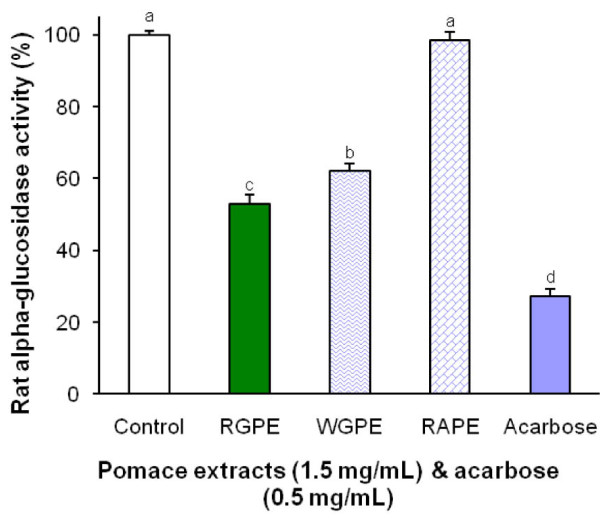
**The inhibitory effects of the grape and apple pomace extracts on rat intestinal α-glucosidase activity**. The α-glucosidase extract was prepared from rat intestinal powder. The enzyme activity was determined by measuring *p*-nitrophenol released from pNPG at 405 nm. The reaction was conducted at 37°C for 75 minutes. Results are expressed as mean ± standard deviation. Bars with different letters are significantly different (*P *< 0.05).

**Figure 3:**
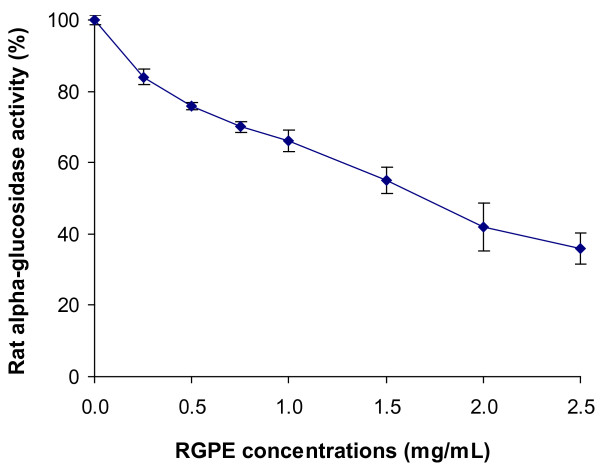
**Dose-dependent inhibition of rat intestinal α-glucosidase activity by RGPE**. The RGPE was tested with concentrations ranging from 0 to 2.5 mg/mL. Results are expressed as mean ± standard deviation.

Figure 2 shows the effects of the pomace extracts on rat intestinal α-glucosidase, both RGPE and WGPE at 1.5 mg/mL in the reaction mixtures exerted significant inhibitory activity on the enzyme. The RGPE inhibited the enzyme activity by 47% and the inhibition potency was significantly higher than the WGPE which inhibited α-glucosidase activity by 39% (*P *< 0.05). As a comparison, the RAPE at 1.5 mg/mL did not exert any effect on rat α-glucosidase while acarbose at 0.5 mg/mL inhibited the enzyme by 73%. A dose-dependent inhibition of rat intestinal α-glucosidase by the RGPE was observed in the reaction with concentrations ranging from 0-2.5 mg/mL (Figure 3). The RGPE inhibited over 64% of the rat α-glucosidase activity at a concentration of 2.5 mg/mL. The IC_50 _of the RGPE were determined as 1.63 mg/mL.

### Inhibition of postprandial hyperglycemia by the acute intake of RGPE in STZ-induced diabetic mice

**Figure 4:**
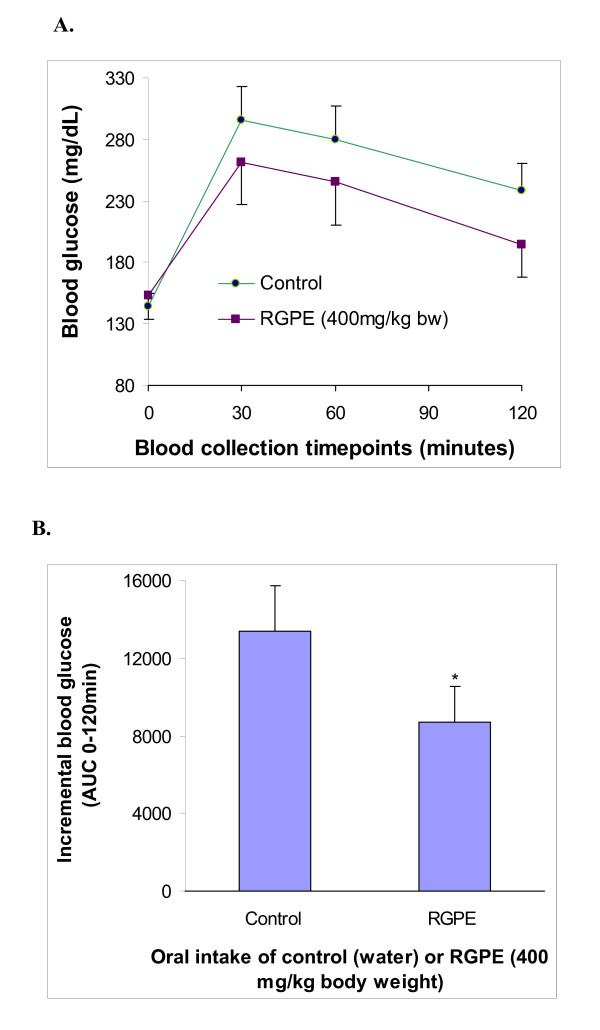
**The inhibitory effect of the RGPE oral intake on postprandial hyperglycemia in STZ-induced diabetic mice after starch challenge**. The fasted STZ-induced diabetic mice were administered with 100 μL of either vehicle or RGPE solution (400 mg/kg, bw) by gavage. After approximately 15 min, 100 μL of potato starch solution (2 g/kg, bw) was administered and blood was collected from tail vein at 0, 30, 60, and 120 min to determine blood glucose levels. **A**. The glycemic response curve in diabetic mice after starch challenge. **B**. The incremental AUC_0-120 min _in diabetic mice after starch administration. *, *P *< 0.05 vs. control (n = 8).

The oral administration of the RGPE (400 mg/kg bw) suppressed postprandial hyperglycemia in STZ-induced mice after the starch challenge (2 g/kg bw) (Figure 4A). After extrapolation of the data using the area under the curve (AUC), the overall percent glucose suppression between the control and RGPE administered groups was determined. The RGPE group had a significant 35% reduction in the glucose AUC_0-120 min _compared to the control group (*P *< 0.05) (Figure 4B).

## Discussion

### Phenolic content and antioxidant properties of the pomace extracts

Phenolic compounds have been recognized as important natural antioxidants rich in many kinds of foods and plants. Apple and grape pomace also contain significant but remarkably varied amounts of phenolic compounds [35,47]. Similar to previous pomace antioxidant reports [48,49], the grape and apple pomace extracts in our study exerted significant peroxyl radical (ORAC) and DPPH radical scavenging activities. In our experiments, all the pomace samples were extracted and prepared under the same conditions. We detected that the RGPE exerted the highest TPC and ORAC among the tested pomace extracts, suggesting the RGPE is one of the better pomace resource of natural antioxidants.

### *In vitro *yeast and rat intestinal α-glucosidase inhibition by the pomace extracts

Yeast α-glucosidase is readily available in a pure form and has been widely used for anti-diabetes nutraceutical and medicinal investigations as a model for screening potential inhibitors [13,20,42,44-46]. We showed that both the grape pomace extracts but not the apple pomace extract significantly inhibited yeast α-glucosidase and their inhibitory activity were stronger than acarbose, the commercial α-glucosidase inhibitor (Figure 1). It is known that α-glucosidase is sensitive to the pH and the pH of the sample extracts may affect the enzyme activity. In this study, the pomace extracts were diluted from the stock 20 mg/mL DMSO solution with 0.1 M phosphate buffer (pH 6.8) and the dilution factor was more than 1000 fold, thereby eliminating the possible effect of the sample pH. Therefore, the significant inhibitory effects of RGPE and WGPE on α-glucosidase are attributable to the unique compounds contained in the extracts.

To determine whether the inhibition is specific, we further evaluated the effects of the RGPE and WGPE on other digestive enzymes including pancreatic α-amylase and lipase. However, both the RGPE and WGPE showed no significant inhibition on the two digestive enzymes (data not shown). Both α-amylase (E.C. 3.2.1.1) and α-glucosidase (EC 3.2.1.20) belong to the glycoside hydrolase family 13 and share a common reaction mechanism and several short conserved sequences [50]. Non-specific inhibitors often inhibit both enzymes due to their structural similarities. However, our results showed that the grape pomace extracts (both the WGPE and RGPE) significantly inhibited α-glucosidase but not α-amylase, suggesting that they are likely specifically targeting α-glucosidases and their inhibiting mechanism may differ from that of acarbose, which inhibits both α-amylase and α-glucosidase. Acarbose has been used for diabetes treatment but this agent has been problematic due to associated adverse gastrointestinal (GI) side effects as a result of its non-specific inhibition of α-amylase, causing excessive accumulation of undigested carbohydrates in the large intestine [3], [51]. Therefore, specific α-glucosidase inhibitors may provide a novel antidiabetic effect and at the same time fewer GI side effects than currently available inhibitors. As such, grape pomace extracts that can specifically inhibit α-glucosidases may have utility for diabetes management with reduced side effects.

It should be noted that although yeast α-glucosidase has been commonly used for anti-diabetic nutraceutical and medicinal investigations, it is not biologically relevant to the mammalian α-glucosidases. In an effort to further establish the effectiveness of the RGPE and WGPE on mammalian α-glucosidase, we further carried out the enzyme inhibition tests using the mammalian α-glucosidases prepared from rat intestinal acetone powder. Indeed, both the grape pomace extracts significantly inhibited rat intestinal α-glucosidase (Figure 2). The RGPE exerted stronger inhibitory activity than the WGPE and the trend was consistent with the results detected from the yeast α-glucosidase assay (Figure 1B). The IC_50 _of the RGPE was further determined to be 1.63 mg/mL which was comparable to other natural α-glucosidase inhibitors such as oolong tea extract (IC_50 _= 1.34 mg/mL) and green tea extract (IC_50 _= 0.735 mg/mL) [52]. Oral administration of the green tea extract (300 mg/kg bw for 4 weeks) was found to significantly reduced fasting blood glucose (by 54%) in STZ-induced diabetic rats [53]. Our results revealed that the grape pomace extracts were more effective in inhibiting yeast α-glucosidase than rat intestinal α-glucosidases. This could be because the extracts selectively inhibit specific α-glucosidases instead of all the enzymes, so the unaffected α-glucosidases in the complex can still hydrolyze pNPG to develop color. Thus, to better understand the inhibition of GSE on mammalian α-glucosidases, further research is needed to assess the inhibiting action of RGPE on specific rat α-glucosidases.

### Inhibition of postprandial hyperglycemia by the acute intake of RGPE in STZ-induced diabetic mice

The potent *in vitro *inhibition of intestinal α-glucosidase by RGPE prompted us to assess whether RGPE can also inhibit α-glucosidase *in vivo*, limit or delay starch digestion and absorption, and subsequently reduce postprandial glycemic response. We induced diabetes in 6-8 week old male C57BL/6J mice by STZ through intraperitoneal injection. The diabetic mice were fasted and orally gavaged with the RGPE solution followed by a potato starch meal. The acute intake of the RGPE suppressed the postprandial blood glucose AUC_0-120 min _in STZ-induced diabetic mice by 35% (*P *< 0.05) (Figure 4B). Although the inhibiting effect of the RGPE intake did not achieve statistical significance in the tested individual time points, there was a noticeable trend indicating the potential glucose lowering effect of the RGPE as compared to the control group. The mechanism by which these results evolved are proposed to be a consequence of RGPE inhibiting the metabolism of carbohydrates by the inhibition of brush border α-glucosidase in the small intestine, similar to that of acarbose [54]. Based on the results obtained with intestinal alpha-glucosidase, it should be expected that in vivo acarbose is more effective then RGPE in reducing postprandial hyperglycemia. Nevertheless, the *in vivo *results suggest that the RGPE may exert potential anti-diabetic effect by suppressing postprandial blood glucose through the novel inhibition of intestinal α-glucosidase.

## Conclusion

To our knowledge, this was the first report that the grape pomace extract exerted significant anti-postprandial hyperglycemic effect, suggesting that grape pomace could be a valuable food derived bioresource that is rich in antioxidants and anti-hyperglycemic compounds. These dual bioactive attributes derived from the grape pomace could play a complementary and alternative role in managing the poorly regulated blood glucose levels and oxidative stress associated with Type 2 diabetes.

## Competing interests

The authors declare that they have no competing interests.

## Authors' contributions

SH performed the animal study, LZ, JL, and KZ conducted antioxidant and enzyme measurements, XJ performed *in vitro *enzyme tests, SS and CC collected and analyzed data, KZ designed the experiments and wrote the manuscript. All authors read and approved the final manuscript.
